# Immune Checkpoint Inhibitor-Induced Diabetes Mellitus—A Brief Review and Three Case Reports

**DOI:** 10.3390/jcm14186620

**Published:** 2025-09-19

**Authors:** Marius-Lucian Mitrache, Aura-Diana Reghina, Iulia-Simona Stoian, Simona Fica

**Affiliations:** 1 Department of Endocrinology, Carol Davila University of Medicine and Pharmacy, 010825 Bucharest, Romania; aura.reghina@umfcd.ro (A.-D.R.); iulia-simona.soare@umfcd.ro (I.-S.S.);; 2 Department of Endocrinology, Elias University Emergency Hospital, 050474 Bucharest, Romania

**Keywords:** diabetes mellitus, immune checkpoint inhibitors, autoimmune, immune-related adverse events, immunotherapy

## Abstract

Immune checkpoint inhibitors (ICIs) have emerged as the cornerstone of treatment in a broad range of neoplasms, but at the cost of several types of immune-related adverse events (irAEs), some of which can also involve the endocrine system. Among those, ICI-induced diabetes mellitus (ICI-DM), while generally considered rare, has been growing in incidence in the past years. While this growth mostly reflects the expanding indications for the use of ICI, several other risk factors have also been described, but they have not been fully characterized. As with the majority of endocrine irAEs, once an endocrine loss of function occurs, it is usually irreversible and requires lifelong substitution therapy. However, the uniqueness of ICI-DM among those stems from the fact that its clinical presentation is usually acute, often life-threatening, and sometimes requires at least brief cessation of immunotherapy. In this paper, we report three cases of ICI-DM and provide a review of the literature regarding this topic, while presenting the real-world clinical lessons we learned from managing these cases, which can prove valuable for both oncologists and endocrinologists.

## 1. Introduction

### 1.1. Background

**Figure 1:**
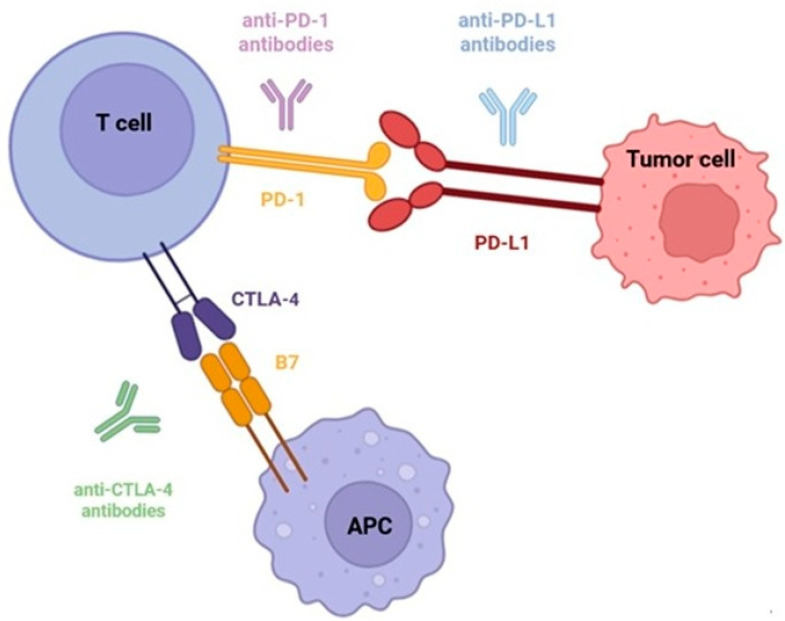
Mechanisms of action of the main ICl agents currently in use. **PD-L1** expressed by tumor cells binds to **PD-1** on activated T cells, inhibiting cytotoxic T cell activity. B7 expressed by APCs acts as a co-inhibitory signal through CTLA-4 located on T cells. Blocking either CTLA-4 or PD-1/PD-L1 using specific antibodies results in T cell activation and cytotoxic antitumoral effect.

Immune checkpoint inhibitors (ICI) emerged as a novel therapy in oncology, providing important outcomes in multiple types of both solid and hematological neoplasms, such as melanoma, renal carcinoma and lung tumors [1,2,3,4]. The agents currently in use can target either Programmed Death-1 Receptor (PD-1) and Cytotoxic T Lymphocyte Associated Protein-4 (CTLA-4) located on T cells, or Programmed Death Ligand-1 (PD-L1) located on tumor cells, thus counteracting the main mechanisms by which tumor cells exploit the immune response and generate immune tolerance towards tumor cells. Chiefly, by pharmacologically counteracting these mechanisms, cytotoxic T cells are driven towards generating a sustained immune response, and their effect increases against the tumor. These mechanisms are illustrated further in Figure 1. However, both PD-1 and CTLA-4 are located on activated T cells, and their expression and interaction with their respective ligands are fundamental in immune tolerance [5]. Thus, targeting these mechanisms can lead to a larger pool of activated and autoreactive cytotoxic T cells, triggering autoimmune complications collectively known as immune-related adverse events (irAEs) [6,7,8]. Endocrine irAEs can vary from the milder ones, such as hypothyroidism to severe or potentially life threatening, such as adrenal insufficiency, hypophysitis or diabetic ketoacidosis, the latter of which may also require ICI cessation as part of their management, combined with adequate hormone replacement therapy, depending on the affected endocrine gland [9,10,11]. Although endocrine irAEs such as thyroiditis, hypophysitis and diabetes mellitus are quite rare compared to others, their uniqueness stems from their irreversibility, which is especially true in endocrine insufficiencies [11,12]. It has long been recognised that the occurrence of vitiligo in patients receiving ICIs for melanoma is an indication of a good therapeutic response [13]. By extent, multiple other studies have correlated the presence of irAEs with an increased progression-free survival [14,15].

### 1.2. Epidemiology and Risk Factors

ICI-induced diabetes mellitus (ICI-DM) occurs rarely, having been reported in approximately 1% of patients [8,12], and almost exclusively in those receiving anti PD-1 or anti PD-L1 agents. Moreover, CTLA-4 expression on pancreatic beta cells has not been reported to date, potentially explaining this observation.

The first report of ICI-DM came in 2012 [16]. In this study, 207 patients with metastatic cancers, most of which included non-small cell lung carcinoma, melanoma, colorectal and renal cell carcinoma received anti-PD-L1 antibodies and both response rates and adverse events were evaluated. Out of all the patients, one developed ICI-DM. Moreover, a case series published in 2015 [17] reported new-onset diabetes mellitus in five patients receiving anti-PD-1 agents either alone or in combination with other cancer treatments. Most of them had acute clinical presentations, and all of them had low or undetectable C-peptide. Three out of five also tested positive for islet autoimmunity, all characteristics pointing towards a fulminant loss of insulin production which was, most likely, autoimmune in nature, an event which is quite rare in this particular age group. Of particular note, some patients in this case series also had a personal history of other autoimmune diseases, two associating autoimmune thyroiditis and one psoriasis. Indeed, other studies have established a link between preexisting autoimmune disease and the risk of developing irAEs in patients receiving ICI therapy [18]. Other patient-specific risk factors include age and gender [19], as younger individuals appear to be at risk for developing irAEs that are more frequent or severe, whereas men receiving anti-PD-1/PD-L1 and women receiving anti-CTLA-4 antibodies seem to be more predisposed [20,21]. Further studies have also established positive correlations between the development of irAEs and several other factors such as a higher body mass index independent of metabolic complications [22], poor ECOG performance status [23], smoker status [24,25], sarcopenia [26], and even family history of autoimmune disease [27].

A large retrospective single-center study which included 8199 patients who received ICI therapy also explored the clinical characteristics and potential predictors for developing ICI-DM [28]. Interestingly, this study reported a much higher incidence of ICI-DM than previously described, as even after adjusting for other factors that could cause hyperglycemia, such as treatment with glucocorticoids or immunosuppressants, 713 patients (8.7%) developed this complication. This may in part be explained by the fact that this study assessed a large number of patients and included milder clinical manifestations of ICI-DM, whereas other studies mainly focused on acute presentations, chiefly diabetic ketoacidosis. However, as acknowledged by the authors, C-peptide and islet autoimmunity panels were only documented in a small fraction of the patients diagnosed, which makes it rather hard to explore the pathogenesis in this group. Intriguingly, the authors also reported a correlation between the presence of either hypertension or dyslipidemia and the development of ICI-DM in the studied population.

### 1.3. Pathogenesis

The main mechanism of ICIs revolves around amplifying the immune response on tumor cells. Chiefly, CTLA-4 and PD-1 located on T cells, and PD-L1 located on tumor cells act as inhibitors of the immune response, generating T cell apoptosis and depletion, in addition to a decreased cytotoxic activity on tumor cells [29,30]. However, these same mechanisms are pivotal in supporting immune self-tolerance. On one hand, CTLA-4 is expressed on regulating T cells and in essence it acts as a negative regulator of T-cell mediated immune response, by binding available ligands from antigen-presenting cells, and by inducing the production of inhibiting cytokines such as transforming growth factor-β [29]. Its fundamental role in immune homeostasis is further supported by several early experimental studies, which have shown that CTLA-4-deficient mice suffer generalised lymphocyte activation and multiorgan infiltration with lymphocytes, dying from fatal autoimmune disease within weeks from birth [31,32], whereas deletion of CTLA-4 in adult mice resulted in a condition similar to Sjögren’s syndrome [33], thus suggesting more complex underlying mechanisms. On the other hand, the PD-1 and PD-L1 system is mainly involved in the initial steps of immune activation, limiting self-reactive T cells [30,34]. Experimental data shows that PD-1-deficient mice develop autoimmune cardiomyopathy [35], dacryoadenitis [36], lupus-like glomerulonephritis [37] and diabetes mellitus [38,39], whereas PD-L1 deficiency in mice leads to encephalomyelitis [40]. Studies on mice have also explored the role of immune checkpoints in the development of diabetes mellitus, proving that the blockade of PD-1 or PD-L1 was associated with fulminant diabetes in non-obese diabetic mice, whereas this was not correlated with the blockade of PD-L2 [38]. Furthermore, injecting anti-PD-1/PD-L1 agents in non-obese diabetic mice was positively correlated with the development of diabetes mellitus when compared to controls [41,42].

In humans, CTLA-4 deficiency leads to a clinical phenotype consisting of autoimmune pancytopenia, encephalomyelitis, thyroiditis and diabetes mellitus [43]. PD-L1 deficient humans exhibit neonatal-onset type 1 diabetes mellitus and autoimmune thyroiditis, whereas PD-1 deficiency is more severe, presenting with fatal autoimmune pneumonitis in childhood [44,45]. Additionally, several other genetic factors have been described in ICI-DM. Patients with HLA-DR3-DQ2 and HLA-DR4-DQ8 haplotypes have a higher chance of developing ICI-DM when treated with anti-PD-1 agents [46], although genetic susceptibility appears to be different in other ethnic groups, as another study [47] incriminated HLA-DR9 and HLA-DRB1*0405*03-DQB1*0401, while CTLA-4 and PD-1 gene polymorphisms also appear to play a role in the development of several autoimmune diseases, including type 1 diabetes mellitus [48,49].

Germline genetic mutations could also play a role in the pathogenesis of ICI-DM, as pointed out by a study from 2023 [50], which described a higher prevalence of CEMIP2 and NLRC5 gene mutations in patients who developed ICI-DM. The same study also identified several genes that were co-expressed in both tumor and normal pancreatic tissue which could act as shared antigens during ICI treatment, among which G6PC and ORM1 are of particular note.

To briefly summarize the existing evidence, known predictors for developing ICI-DM are mostly clinical and easy to identify, and include personal or family history of autoimmune diseases (either endocrine or otherwise), younger age, higher BMI, male gender, poor ECOG performance status, and anti-PD-1/PD-L1 or combined anti-CTLA-4/PD-1 therapy. Available data regarding genetic risk factors is rather conflicting and it is also difficult and expensive to implement thorough genetic screening protocols on a larger scale in clinical practice, although this may prove useful in future investigations.

### 1.4. Clinical Features and Management

Since PD-L1 is naturally expressed in beta cells, ICI-DM most likely occurs due to autoimmune destruction of these cells, resulting in total or near-total insulin deficiency, thus explaining the clinical similarities between ICI DM and type 1 diabetes mellitus [51]. ICI-DM usually occurs early after the initiation of treatment and most cases present with symptoms suggesting severe hyperglycemia, such as polyuria, polydipsia and significant weight loss, while sometimes an acute presentation with diabetic ketoacidosis can occur. Marked hyperglycemia is confirmed, with blood glucose levels as high as 1000 mg/dL found in most patients, whereas HbA1c levels is often only mildly elevated at diagnosis, suggesting an acute loss of insulin secretion. C peptide is frequently very low or undetectable, and autoimmune panel consisting of anti-insulin, anti-glutamic acid decarboxylase, anti-islet cell antigen can be positive [51,52,53].

The management of ICI-DM should start with the early recognition of symptoms suggestive of hyperglycemia, of which patients receiving ICI therapy and their families should be made aware. It is also essential for oncologists to routinely check plasma glucose HbA1c levels in their patients receiving ICI. Collaboration with an endocrinologist is key and the treatment plan should be guided by the severity of the clinical presentation. Patients presenting with diabetic ketoacidosis should promptly receive intravenous insulin in combination with fluid and electrolytes, while for milder presentations subcutaneous insulin injections are preferred [54]. While glucocorticoids in high doses are standard practice in some ICI-induced irAEs, such as hypophysitis, they are not usually indicated in the management of ICI-DM, as they do not revert it and can further worsen glycemic control [55].

Although endocrine irAEs, including ICI-DM, are generally considered to be irreversible, there is interestingly one case report of spontaneous recovery from insulin dependence [56]. Of note, the patient in question also had a history of ICI-associated secondary adrenal insufficiency, hinting towards a predisposition to developing irAEs, and a protective allele against type 1 diabetes mellitus.

We report three cases of newly diagnosed insulin-dependent diabetes mellitus in patients receiving ICIs as treatment for cancer.

## 2. Materials and Methods

### 2.1. Case Report 1

The first case is that of a 53-year-old male patient diagnosed with subungual melanoma of the thumb, metastatic to the liver and lungs for which combination immunotherapy with a CTLA-4 inhibitor and a PD-1 inhibitor was initiated in February 2019. Other than his gender and the treatment regimen itself, this patient had no other known risk factors for developing ICI-DM.

Following two treatment cycles, the patient developed rapid weight loss, polyuria and polydipsia over the course of two weeks. Primary evaluation showed a capillary glucose level of 757 mg/dL, glucosuria and metabolic acidosis, and the patient was quickly admitted to the Endocrinology Department. Lab tests revealed a blood glucose level of 470 mg/dL and a glycated hemoglobin of 8.1%. Islet cell autoantibodies, glutamic acid decarboxylase autoantibodies and insulin autoantibodies were positive, and C peptide was undetectable, thus proving the autoimmune etiology.

The patient received hydration, intravenous and subcutaneous insulin and clinically stabilised over the next days, eventually being discharged and receiving basal-bolus insulin therapy. He continued ICI therapy, leading to a complete response.

### 2.2. Case Report 2

A 58-year-old female patient was diagnosed with right arm melanoma on the deltoid region in October 2022, for which surgery was performed. Of note was the patient’s personal history of chronic autoimmune thyroiditis and primary hypothyroidism, for which she received thyroid replacement therapy in the form of 100 micrograms levothyroxine daily. Follow-up one month later showed a recurrence of the primary tumor and dissemination to the left axillary lymph nodes. Repeat surgery for the melanoma was performed in November, and combined immunotherapy with an anti-CTLA-4 and an anti-PD-1 agent was initiated in March 2023.

Two months after the initiation of ICI therapy, the patient developed grade 3 gastrointestinal toxicity with severe diarrhoea and dehydration, leading to the temporary discontinuation of treatment. However, one week later she presented to the Emergency Department with refractory headache, photophobia and nausea. Emergency computed tomography of the head excluded stroke, infection and brain metastasis, but raised to suspicion of hypophysitis, which was later strengthened by magnetic resonance imaging describing the mild enlargement of the pituitary gland. Hormone panel was consistent with secondary adrenal insufficiency, showing low cortisol and ACTH levels. Thus, as per the guidelines available at that time, high dose glucocorticoid therapy was initiated, leading to the resolution of the symptoms over the following days. As such, the patient was discharged with glucocorticoid replacement therapy, and the multidisciplinary team managing the case also decided to resume ICI therapy.

Four months later, in September 2023, the patient presented with rapid weight loss (approximately 10 kg), polyuria and polydipsia. Capillary glucose levels were 314 mg/dL, while further blood tests showed hyperglycemia (450 mg/dL) and a HbA1c level of 8.2%, thus confirming the diagnosis of diabetes mellitus. Further autoantibody tests showed positivity only for anti-GAD antibodies, but peptide C levels were undetectable, suggesting an autoimmune cause and the lack of insulin secretory capacity of beta cells, which were considered hallmarks of ICI-DM in this context. Intravenous and subcutaneous insulin therapy was initiated, and the patient was later discharged with a basal-bolus insulin regiment. Apart from the combined immunotherapy used, this patient had a known history of autoimmune thyroiditis prior to ICI initiation, and also developed hypophysitis over the course of treatment, both of which can be considered potential risk factors for her developing ICI-DM.

Following this, the team managing the case decided against the continuation of ICI therapy, instead opting for radiotherapy, and after fifteen weeks the patient had a complete response.

### 2.3. Case Report 3

A 43-year-old male patient was diagnosed with clear cell renal carcinoma metastatic to the lungs in March 2024. Total nephrectomy was performed in the same month, and an anti-PD-1 agent was initiated in April 2024. Following eight treatment cycles, the patient developed autoimmune thyroiditis and primary hypothyroidism, for which levothyroxine substitution therapy was prescribed, while also being a potential predictor for developing ICI-DM.

After six more treatment cycles, evaluation at the patient’s general practitioner office showed a capillary glucose level of 455 mg/dL, while of particular note was his lack of symptoms suggestive of hyperglycemia. Nonetheless, capillary glucose levels were repeatedly high, leading to the patient’s referral to our department. On admission, lab tests revealed a fasting plasma glucose of 469 mg/dL, glucosuria, and a glycated hemoglobin of 10.4%. The patient also had a TSH of 52 uIU/mL, and cortisol and ACTH levels were normal. C peptide was undetectable, suggesting beta cell insufficiency, but the autoantibody panel consisting of anti-insulin, anti-GAD, anti-IA2, and anti-ZnT8 antibodies was negative.

The patient received insulin therapy, leading to glycemic improvement over the following days, and he was discharged on a basal bolus insulin regimen. The patient is still alive and continuing anti-PD-1 therapy.

## 3. Discussion

While having a low incidence among irAEs, ICI-DM can be life threatening due to the sudden loss of insulin production in pancreatic beta cells [17,57]. Thus, patients can often have acute presentations, characterized either by diabetic ketoacidosis, which have been reported to occur in more than half of cases, or severe cardinal symptoms of diabetes mellitus [51,52,53], such as important weight loss, polyuria, polydipsia, and fatigue, which is consistent with our reported cases, except the last one.

While a higher body mass index was previously reported as being positively correlated with both a better response to ICI treatment and the development of irAEs [22], the effect of adipokines is less understood. Adiponectin was found to have a positive effect on reducing ICI-induced colitis in mice models [58], while lower circulating leptin levels were found in patients with irAEs that received anti-PD-1 antibodies [24]. The role of other less studied adipokines is definitely an avenue for further research.

Patients usually present with marked hyperglycemia, often more than 800–1000 mg/dL, but only mildly elevated HbA1c levels, further suggesting the near-total and acute loss of insulin production. This is also corroborated by low or undetectable C peptide levels, and positivity of islet autoimmunity panels, found in about half of the cases [51,52,53,59]. This pattern was consistent in all of our three cases, suggesting lack of insulin secretion.

The latency between ICI initiation and ICI-DM diagnosis is variable, having been reported as early as days or as late as years from the first cycle. However, median time to diagnosis is between 7 and 17 weeks, and a faster onset has been reported in patients with PD-1 or PD-L1 and CTLA4 combination therapy, and in patients with positive islet autoimmunity [60,61,62]. This is a pattern consistent with our first case, whereas in our second and third cases, ICI-DM occurred later over the course of treatment. As previously addressed, autoimmune toxicity, such as patients with melanoma developing vitiligo while treated with ICI, may be associated with improved cancer-specific mortality, signalling cytotoxic T cell activity. As such, it is likely that developing other types of irAEs, such as autoimmune thyroiditis or ICI-DM has the same significance. Two of our three cases adhered to this pattern, but we admit, however, that one cannot draw any relevant conclusions based on this limited number of cases. Furthermore, in the case of the second patient, while having multiple autoimmune toxicities, the response to ICIs was unsatisfactory, thus leading to the team’s decision to switch therapy.

Although it is among the rarest irAEs, ICI-DM has been growing in incidence over the past years, probably owing to the expanding indications of immunotherapy. However rare it might be, considering its potential severity and life-threatening nature, clinicians should be aware of it and collaborate as a part of a multidisciplinary team when managing ICI-DM.

As illustrated by the three cases we reported, the main challenge arises from the fact that hyperglycemia is not always clinically evident. The same applies to other endocrine autoimmune toxicities of ICI, and this is the reason why, in our opinion, screening for these should not be guided exclusively in patients with a high degree of clinical suspicion. A skilled and inquisitive endocrinologist, well-versed in the often subtle signs that point towards an endocrine dysfunction, should be a key part of the team managing ICI patients, as demonstrated by our experience. Potential risk factors should always be reviewed, and it is fortunate that most of them are clinical and evident during patient history and clinical examination, as earlier discussed. Well established protocols, which also take local population characteristics into consideration, could further be of aid. We suggest that patients receiving ICI therapy should undergo systematic screening for hyperglycemia based at least on measuring fasting capillary glucose levels before each treatment cycle, considering that it is quite inexpensive, and further testing can be adjusted based on this. Additional basic endocrine screening consisting of thyroid function tests could also be employed, based on the fact that thyroid dysfunction is not always clinically evident. It is important to note that, while the literature agrees that most endocrine autoimmune events, including ICI-DM, occur early over the course of treatment, one should keep in mind that these events do not adhere to any set of rules or guidelines. As shown in our second case, patients can develop this complication as late as six months after initiation of therapy. Thus, we suggest continuing clinical follow-up and screening directed by an endocrinologist, even after several uneventful treatment cycles. If they occur, endocrine irAEs fortunately do not require permanent discontinuation of immunotherapy, although a temporary cessation may be needed. In ICI-DM, a provisional interruption of therapy is required when patients are ketoacidotic. After the fluid and electrolytes balance has been settled, and patients have received adequate insulin therapy, it is reasonable to consider resuming ICI treatment.

## 4. Conclusions

The heterogeneity of ICI-DM is well reflected in its multiple proposed pathogenic mechanisms, potential clinical and biochemical predictors, and variable clinical presentations, aspects which we briefly covered in this study. Given the narrative approach of this review, it has, of course, several limitations, the most important of which is the lack of a systematic approach. We also recognise that the low number of cases we reported leads to limited generalizability, but this is often true for all rare diseases or clinical outcomes, which is why multicentered efforts with a larger pool of cases should further address the issue. Moreover, we did not conduct genetic profiling in our patients, which could have proved useful since, as discussed earlier, certain haplotypes and genes can carry a risk in ICI-DM development.

As previously mentioned, patients with a higher body mass index appear to be at risk for developing irAEs, but most of the data supporting this originates from studies that used anthropometric parameters. We suggest that future large-scale studies should also make use of more complex and novel evaluations, such as determining body composition using dual-energy X-ray absorptiometry, which could potentially uncover further missing links.

Finally, it is important to acknowledge that in real-world clinical practice, most patients receiving ICI therapy who are referred to the endocrinologist most likely have an already established endocrine irAE, which can lead to selection bias in studies, but this can be prevented if the team managing ICI patients has a dedicated endocrinologist. To further understand all facets of endocrine irAEs in ICI-receiving patients, perhaps future studies should have a more systematic approach, which includes full endocrine evaluation before the initiation, and at set intervals while receiving therapy. Although this would definitely be a costly endeavor, and would require clockwork collaboration between departments, it could prove fundamental in further establishing the key aspects of ICI-DM, ultimately serving in improving the standard of care in oncological patients.

## Data Availability

No new data were created or analyzed in this study.

## Abbreviations

The following abbreviations are used in this manuscript:

ICI	Immune checkpoint inhibitor
PD-1	Programmed Death-1 Receptor
CTLA-4	Cytotoxic T Lymphocyte Associated Protein-4
PD-L1	Programmed Death Ligand-1
irAEs	immune-related adverse events
ICI-DM	ICI-induced diabetes mellitus
